# Risk of Community-Acquired Pneumonia with Outpatient Proton-Pump Inhibitor Therapy: A Systematic Review and Meta-Analysis

**DOI:** 10.1371/journal.pone.0128004

**Published:** 2015-06-04

**Authors:** Allison A. Lambert, Jennifer O. Lam, Julie J. Paik, Cesar Ugarte-Gil, M. Bradley Drummond, Trevor A. Crowell

**Affiliations:** 1 Department of Medicine, Division of Pulmonary and Critical Care, Johns Hopkins University, Baltimore, MD, United States of America; 2 Department of International Health, Johns Hopkins University, Baltimore, MD, United States of America; 3 Department of Medicine, Division of Rheumatology, Johns Hopkins University, Baltimore, MD, United States of America; 4 Instituto de Medicina Tropical Alexander Von Humboldt, Universidad Peruana Cayetano Heredia, Lima, Perú; 5 Department of Medicine, Division of Infectious Diseases, Johns Hopkins University, Baltimore, MD, United States of America; Cleveland Clinic, UNITED STATES

## Abstract

**Background:**

Proton-pump inhibitors (PPIs) are among the most frequently prescribed medications. Community-acquired pneumonia (CAP) is a common cause of morbidity, mortality and healthcare spending. Some studies suggest an increased risk of CAP among PPI users. We conducted a systematic review and meta-analysis to determine the association between outpatient PPI therapy and risk of CAP in adults.

**Methods:**

We conducted systematic searches of MEDLINE, EMBASE, CINAHL, Cochrane Central Register of Controlled Trials, Scopus and Web of Science on February 3, 2014. Case-control studies, case-crossover, cohort studies and randomized controlled trials reporting outpatient PPI exposure and CAP diagnosis for patients ≥18 years old were eligible. Our primary outcome was the association between CAP and PPI therapy. A secondary outcome examined the risk of hospitalization for CAP and subgroup analyses evaluated the association between PPI use and CAP among patients of different age groups, by different PPI doses, and by different durations of PPI therapy.

**Results:**

Systematic review of 33 studies was performed, of which 26 studies were included in the meta-analysis. These 26 studies included 226,769 cases of CAP among 6,351,656 participants. We observed a pooled risk of CAP with ambulatory PPI therapy of 1.49 (95% CI 1.16, 1.92; I2 99.2%). This risk was increased during the first month of therapy (OR 2.10; 95% CI 1.39, 3.16), regardless of PPI dose or patient age. PPI therapy also increased risk for hospitalization for CAP (OR 1.61; 95% CI: 1.12, 2.31).

**Discussion:**

Outpatient PPI use is associated with a 1.5-fold increased risk of CAP, with the highest risk within the first 30 days after initiation of therapy. Providers should be aware of this risk when considering PPI use, especially in cases where alternative regimens may be available or the benefits of PPI use are uncertain.

## Introduction

Community-acquired pneumonia (CAP) is a common diagnosis associated with substantial morbidity and healthcare expenditure. In 2006 alone, 4.2 million ambulatory care visits for CAP occurred in the United States [1]. Medicare data from 2007–2008 indicated a 30-day mortality ranging from 3.8 to 8.5% depending on severity of disease [2]. Annual healthcare costs incurred by patients with CAP are estimated to be approximately $13 billion among Medicare fee-for service patients [2]. Implementation of guidelines for antibiotic selection [3, 4] and administration of pneumococcal vaccination [5, 6] have been shown to reduce CAP incidence, morbidity and mortality. Identification and avoidance of medications associated with an increased risk of CAP could further reduce CAP incidence.

Proton pump inhibitors (PPIs) are among the most widely prescribed medications. In 2011, omeprazole was the sixth most commonly prescribed medication in the United States with nearly 60 million prescriptions [7]. PPIs have become a 10 billion dollar industry with over 15 million Americans taking these medications, not including over-the-counter usage [8]. Although evidence and guidelines support the use of PPIs for gastroesophageal reflux disease (GERD) [9] and select cases of duodenal and gastric ulcers [10], evaluation of PPI therapy in the ambulatory setting suggests that as few as 35% of patients taking PPIs have an appropriate indication documented [11, 12]. A spectrum of side effects are associated with PPI therapy, including deficiencies of critical vitamins and minerals, *Clostridium difficile*-associated diarrhea infection and hip fracture [13]. CAP has been hypothesized as an additional untoward consequence of PPI therapy [14].

We conducted a systematic review and meta-analysis of observational and randomized studies to determine whether outpatient PPI therapy is associated with increased risk of CAP among adults as compared to no PPI therapy. To identify particularly high-risk patients, we conducted additional analyses to determine the CAP risk stratified by PPI dose, duration of PPI therapy, and age of participant.

## Methods

Our systematic review and meta-analysis was conducted in accordance with the Preferred Reporting Items for Systematic Reviews and Meta-Analyses (PRISMA)[15] and Meta-analysis of Observational Studies in Epidemiology (MOOSE)[16] (PRISMA checklist reported in S1 Table). A written review protocol was not drafted.

### Data Sources and Search Strategy

We performed systematic searches of MEDLINE (via PubMed), EMBASE, CINAHL, Cochrane Central Register of Controlled Trials (CENTRAL), Scopus, Web of Science and ClinicalTrials.gov on February 3, 2014. Our search strings included controlled vocabulary and related keywords for two concepts: pneumonia and acid suppressants (S2 Table). We did not limit our search based on study design, publication year, language or inclusion of human participants. Authors of potentially eligible abstracts, posters or manuscripts were contacted via email to clarify study information and obtain additional data. Approval for use of unpublished data in our analysis and certification of data validity was documented electronically.

### Study Selection

We included in our review studies reporting data to allow the calculation of a measure of risk of CAP among adult participants with and without exposure to outpatient PPI therapy. We followed guidance from the Cochrane Collaboration to include both observational and randomized studies to maximize study, participant and event inclusion into our analysis and to thereby allow the conduct of sensitivity and subgroup analyses [17, 18]. We excluded case reports and case series. We screened systematic reviews for the inclusion of data not published elsewhere and for references not otherwise captured by our search strategy. We excluded studies in which <95% of participants were over 18 years old. We also excluded studies if PPIs were exclusively administered intravenously, in the inpatient setting, peri-procedurally, or as part of *Helicobacter pylori* therapy. CAP cases were identified by the definitions utilized in each included study. We excluded studies in which CAP preceded PPI exposure or in which the temporal relationship was ambiguous.

### Data Extraction

Two authors independently screened studies for inclusion and a third author adjudicated discordant assessments. Title/abstract and full text screening were conducted in a similar fashion; however, specific exclusion reasons were documented only during full text screening. Upon selection of the final group of studies, two authors independently extracted qualitative and quantitative data using a standardized data extraction form adjudicated by a third author. To assess the methodological quality of observational studies, we used a modified version of the Newcastle-Ottawa Scale [19] (S3 Table). We applied this validated tool to characterize participant selection, comparability of populations, and outcome assessment.

### Analyses

The primary outcome of this meta-analysis was incident CAP during treatment with outpatient PPI therapy. Sensitivity analyses examined our primary outcome among studies in which PPI therapy was the single form of gastric acid suppression, studies with our strict definition of CAP that included radiographic confirmation, and studies with lower risk of bias (defined as low risk on ≥4 out of 7 criteria for cohort studies and ≥6 out of 8 criteria for case-control studies using the modified Newcastle-Ottawa Scale). Secondary analyses evaluated the risk of CAP with H2-receptor antagonist (H2RA) therapy and risk of hospitalization for CAP with PPI therapy. Lastly, we examined three subgroups of participant populations in order to more specifically characterize the risk of CAP associated with PPI therapy: those treated with different PPI doses (high dose > 1 defined daily dose [DDD] and low dose PPI ≤ 1 DDD), durations of PPI therapy prior to CAP diagnosis (<1 month, 1–6 months, >6 months), and age categories (<65 years old or ≥65 years old). All subgroup analyses were defined *a priori*.

### Statistical Analysis

We report measures of association extracted from the included studies: odds ratios (ORs), relative risks (RRs) and hazard ratios (HRs), each with a 95% confidence interval (CI). For studies that did not report a measure of association between PPI exposure and CAP but did include the required information, we calculated an unadjusted odds ratio using the Peto method [20].

We generated a funnel plot to visually assess reporting bias and used Egger’s test to assess asymmetry of the funnel plot. In our qualitative analysis, we critically examined the study population, study design, internal and external validity, and exposure and outcome ascertainment and definitions.

To determine the proportion of variability in the effect estimates due to heterogeneity, we calculated the I^2^ statistic [21]. We adhered to the accepted cutoff of 50% to define significant heterogeneity and additionally calculated the associated p-value (via χ^2^ test) [21]. We generated forest plots for each of our exposure-outcome comparisons of interest. Because of significant clinical, methodological and statistical heterogeneity across the included studies, we used a random-effects based model to calculate the weighted mean, variance of the summary effect, and associated 95% confidence interval and p-value. Since pneumonia is a rare clinical event, all effect estimates were assumed to approximate the relative risk and were pooled in meta-analyses. All statistical analyses were conducted using STATA v12 [22].

## Results

### Search Results and Study Characteristics

**Fig 1:**
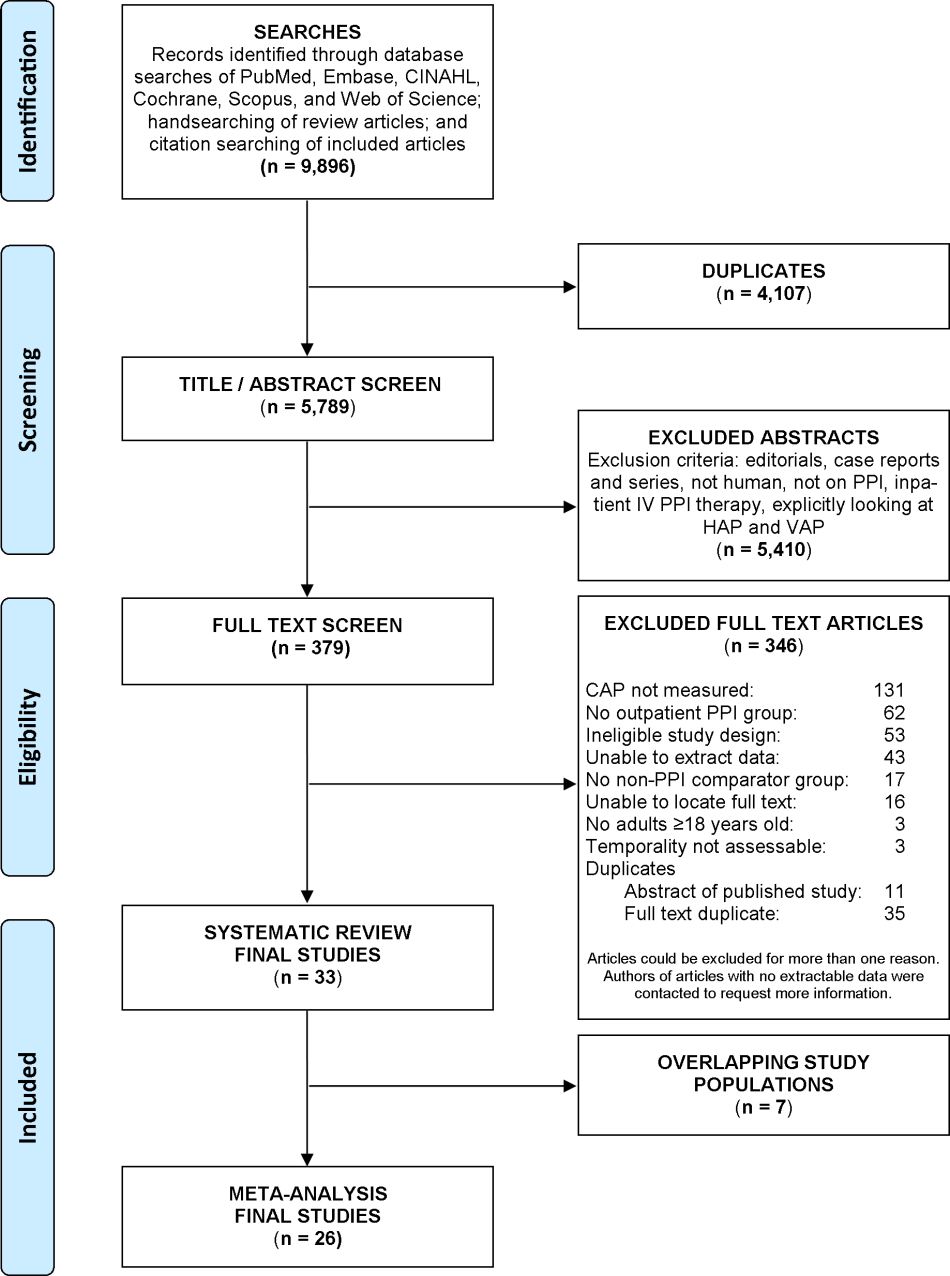
Flow Diagram of Study Selection, adapted from PRISMA Group 2009 Flow Diagram. Studies could be excluded for more than one reason, therefore the sum of exclusion reasons exceeds total studies. Examples of studies lacking extractable data included abstracts without full results (such as abstracts reporting an odds ratio without exposure or outcome definitions) or papers summarizing outcomes qualitatively in the text (such as manuscripts reporting adverse event categories that grouped pneumonia among other events).

Our systematic search identified 5789 unique titles and abstracts. Of these, 5410 studies were excluded based on predetermined eligibility criteria during title/abstract review. The remaining 379 studies underwent full text screening and 33 ultimately met criteria for inclusion in our systematic review [23–55]. Due to overlapping study populations, 26 of these 33 studies were included in our meta-analysis [23–25, 27, 28, 31–40, 43–52, 54]. Of the 346 excluded full texts, the most common reason for exclusion was a lack of CAP assessment (n = 131), followed by absence of an appropriate PPI exposure group (n = 62), and ineligible study design (n = 53). Reasons for exclusion are detailed in the PRISMA flow diagram (Fig 1) [56].

### Systematic Review

A qualitative synthesis of the 33 studies meeting our inclusion criteria is summarized in Table 1 and S4 Table. The majority of studies were of case-control design (n = 18); 10 were cohort studies, 4 randomized controlled trials (RCTs), and 1 case-crossover study. Studies were conducted across the world with the United States and the United Kingdom contributing data to the most studies (11 and 10 studies, respectively). Data collection spanned over 2 decades (1987–2011).

**Table 1:** Qualitative analysis of 32 Studies under Systematic Review.

Author Year, Study Design	Location, Years of Conduct	Source of Cases	CAP setting	CAP Definition	# Cases / # Participants	Acid-suppression	Methods of Ascertainment	Ref.
**Almirall 2008**, Case-Control	Spain, 1999–2000	Patients >14 years old at 64 primary care centers	Inpatient or Outpatient	Medical record review with prescription of antibiotics and new radiological findings suggestive of infiltrate	1336 / 2662	PPI	**PPI:** Questionnaire; **CAP:** Medical, radiographic records	23
**Chen 2013**, Cohort	Taiwan, 1998–2008	CKD patients in Taiwan Health Insurance Research Database	Inpatient	Diagnostic codes (ICD-9)	619 / 8076	PPI	**PPI:** Prescription records; **CAP:** Diagnostic codes (ICD-9)	24
**Dublin 2010**, Case-Control	Washington, USA, 2000–2003	Adults 65–94 years old in Group Health Integrated healthcare delivery system	Inpatient or Outpatient	Diagnostic codes (ICD-9), confirmed by review of radiology and hospital records	1125 / 3360	PPI	**PPI:** Pharmacy database; **CAP:** Diagnostic codes (ICD-9), medical records	25
**Ernst 2012**, Case-Control	United Kingdom, 1997–2009	Anti-parkinsonian drug users 40–89 years old in the General Practice Research Database	Inpatient	Diagnostic codes (ICD-10)	1835 / 17923	PPI	**PPI:** Prescription records; **CAP:** Diagnostic codes (ICD-10)	26
**Filion 2013**, Cohort	Canada, UK, & USA, 1997–2010	New NSAID users ≥40 years old in eight research databases	Inpatient	Diagnostic codes (ICD-10)	5135 / 4238504	PPI	**PPI:** Pharmacy and prescription records; **CAP:** Diagnostic codes (ICD-10)	27
**Gau 2010**, Case- Control	Ohio, USA, 2004, 2006	Rural community hospital admissions ≥65 years old	Inpatient	Discharge diagnosis with radiographic confirmation	194 / 1146	PPI	**PPI:** Medication reconciliation, physician or nursing home notation; **CAP:** Diagnostic codes, medical records	28
**Gulmez 2007**, Case- Control	Funen, Denmark, 2000–2004	Government Patient Registries	Inpatient	Diagnostic codes (ICD-8 & ICD-10)	7642 / 41818	PPI	**PPI:** Pharmacy database; **CAP:** Diagnostic codes (ICD-8 & ICD-10)	29
**Hennessy 2007**, Case-Control	United Kingdom, 1987–2002	Adults ≥65 years old in the General Practice Research Database	Inpatient	Diagnostic codes (Read)	12044 / 60220	PPI	**PPI:** Prescription records; **CAP:** Diagnostic codes (Read)	30
**Hermos 2012**, Case-Control	USA, 1996–2007	New England Veterans Healthcare System	Inpatient or Outpatient	Diagnostic code (ICD-9) and pharmacy record of respiratory antibiotic prescription	1544 / 16984	PPI	**PPI:** Pharmacy database; **CAP:** Diagnostic codes (ICD-9), pharmacy records	31
**Jena 2013**, Cohort	USA, 1997–2007	Adults ≥30 years old in six employer-based insurance plans	Inpatient or Outpatient	Diagnostic codes (ICD-9)	16827 / 54490	PPI	**PPI:** Prescription drug claims database; **CAP:** Diagnostic codes (ICD-9)	32
**Juthani-Mehta 2013**, Cohort	Pennsylvania & Tennessee, USA, 1998–2008	Adults 70–79 years old in the Health, Aging, and Body Composition Study	Inpatient	Medical record review of radiography, respiratory symptoms, physical examination, and diagnostic codes (ICD-9)	193 / 1441	PPI	**PPI:** Patient pill bottles; **CAP:** Diagnostic codes (ICD-9), medical records	33
**Laheij 2003**, Cohort	Netherlands, 2002	Outpatient endoscopy service and surrounding community	Inpatient or Outpatient	Patient report	6 / 405	PPI or H2RA	**PPI:** Self-report via questionnaire; **CAP:** Self-report via questionnaire	34
**Laheij 2004**, Case- Control	Netherlands, 1995–2002	Integrated Primary Care Information (IPCI) project	Inpatient or Outpatient	Medical record review of radiography, microbiology or respiratory symptoms	475 / 5165	PPI	**PPI:** Prescription records, IPCI database; **CAP:** Medical record, IPCI database	35
**Liu 2012**, Case-Crossover	Taiwan, 1998–2007	Adults ≥18 years old with history of stroke and pneumonia hospitalization	Inpatient	Diagnostic codes (ICD-9)	13832 / 13832	PPI	**PPI:** Insurance database; **CAP:** Diagnostic codes (ICD-9)	36
**Long 2013**, Case-Control	USA, 1997–2009	IBD patients <64 years old in the Life Link Health Plan Claims Database	Inpatient or Outpatient	Diagnostic codes (ICD-9) with antibiotic prescription or hospital admission	4856 / 23784	PPI	**PPI:** Outpatient pharmacy claims; **CAP:** Diagnostic codes (ICD-9), pharmacy records	37
**Mastronarde 2009**, RCT	USA, 2004–2008	Adults with poorly controlled asthma enrolled in ALA- ACRC	Outpatient	Not reported	1 / 402	PPI	**PPI:** Randomized per protocol; **CAP:** Adverse event reporting	38
**Meijvis 2011**, Case-Control	Netherlands, 2004–2010	Two teaching hospitals	Inpatient	New infiltrate on a chest radiograph with at least two clinical or laboratory findings consistent with pneumonia	430 / 2150	PPI	**PPI:** Pharmacy database; **CAP:** Medical admission records	39
**Morris 2013**, Cohort	USA, 2009–2011	Kidney transplant recipients at 3 hospitals	Inpatient or Outpatient	Positive sputum culture with medical record review for clinical correlation	4 / 211	PPI	**PPI:** Prescription records; **CAP:** Medical records, sputum culture	40
**Muellerova 2012**, Case-Control	United Kingdom, 1996–2005	COPD patients ≥ 45 years old in General Practice Research Database	Inpatient or Outpatient	Diagnostic codes (Read)	1469 / 8814	PPI	**PPI:** Prescription records; **CAP:** Diagnostic codes (Read)	41
**Myles 2009**, Case-Control	United Kingdom, 2001–2002	Patients >40 years old in The Health Improvement Network (THIN)	Inpatient or Outpatient	Diagnostic codes (Read)	3709 / 25883	PPI	**PPI:** Pharmacy records; **CAP:** Diagnostic codes (Read)	42
**Nielsen 2012**, Case-Control	Denmark, 1997–2009	Patients ≥15 years old in the Danish National Registry of Patients	Inpatient	Diagnostic codes (ICD-8 & ICD-10)	70914 / 780054	PPI	**PPI:** Prescription codes; **CAP:** Diagnostic codes (ICD-8 & ICD-10)	43
**Pasina 2011**, Cohort	Italy, 2008	Patients ≥65 years old admitted at 38 internal medicine wards	Inpatient	Diagnostic codes (ICD-9)	28 / 1332	PPI	**PPI:** Prescription codes; **CAP:** Diagnostic codes (ICD-9)	44
**Quagliarello 2005**, Cohort	New Haven, CT, USA, 2001–2003	Nursing home residents >65 years old	Inpatient or Outpatient	Compatible symptoms with radiographic findings	112 / 613	PPI or H2RA	**PPI:** Nursing home records; **CAP:** Medical & radiographic records	45
**Ramsay 2013**, Cohort	Australia, 2007–2011	Adults ≥ 65 years old, eligible for DVA services	Inpatient	Diagnostic codes (ICD-10)	6775 / 105467	PPI	**PPI:** Prescription records; **CAP:** Diagnostic codes (ICD-10)	46
**Rodriguez 2009**, Case-Control	United Kingdom, 2000–2005	Patients 20–79 years old in The Health Improvement Network (THIN)	Inpatient or Outpatient	Diagnostic codes (Read)	7297 / 17920	PPI	**PPI:** Pharmacy records; **CAP:** Diagnostic codes (Read)	47
**Roughead 2009**, Cohort	Australia, 2002–2006	Patients ≥65 years old with full Veterans' Affairs benefits	Inpatient	Diagnostic codes (ICD-10)	13876 / 185533	PPI	**PPI:** Pharmacy records; **CAP:** Diagnostic codes (ICD-10)	48
**Sarkar 2008**, Case-Control	United Kingdom, 1987–2002	Patients ≥18 years old in General Practice Research Database	Inpatient or Outpatient	Diagnostic codes (Read or Oxford)	80066 / 879947	PPI	**PPI:** Prescription records; **CAP:** Diagnostic codes (Read or Oxford)	49
**Scheiman 2011**, RCT	Europe, Australia, Asia, Africa, Americas, 2007–2008	Aspirin users ≥18 years old with history or risk of peptic ulcer	Inpatient or Outpatient	Not reported	9 / 2426	PPI	**PPI:** Randomized per study protocol; **CAP:** Adverse event reporting	50
**Sugano 2011**, RCT	Japan, 2007–2008	Long-term low-dose aspirin users with history of ulcer	Inpatient	Not reported	1 / 461	PPI	**PPI:** Randomized per study protocol; **CAP:** Adverse event reporting	51
**Sugano 2012**, RCT	Japan, 2007–2009	Long-term NSAID users with history of ulcer	Inpatient	Not reported	6 / 366	PPI	**PPI:** Randomized per study protocol; **CAP:** Adverse event reporting	52
**van de Garde 2006 (Thorax)**, Case-Control	United Kingdom, 1987–2001	Diabetic patients ≥ 18 years old in General Practice Research Database	Inpatient or Outpatient	Diagnostic codes (Read)	4719 / 20041	PPI or H2RA	**PPI:** Prescription records; **CAP:** Diagnostic codes (Read)	53
**van de Garde 2006 (ERJ)**, Case-Control	Netherlands, 1995–2000	Adults >18 years old in the PHARMO record linkage system	Inpatient	Diagnostic codes (ICD-9)	1108 / 4925	PPI	**PPI:** Pharmacy database; **CAP:** Diagnostic codes (ICD-9)	54
**van de Garde 2007 (J HTN)**, Case-Control	United Kingdom, 1987–2001	Diabetic patients ≥ 18 years old in General Practice Research Database	Inpatient or Outpatient	Diagnostic codes (Read)	4719 / 20041	PPI or H2RA	**PPI:** Prescription records; **CAP:** Diagnostic codes (Read)	55

ALA, American Lung Association; ACRC, Asthma Clinical Research Centers; CAP, community-acquired pneumonia; CKD, chronic kidney disease; COPD, chronic obstructive pulmonary disease; CT, Connecticut; DVA, Department of Veterans’ Affairs; H2RA, histamine-2 receptor-antagonist; IBD, inflammatory bowel disease; IPCI, Integrated Primary Care Information; NSAID, non-steroidal anti-inflammatory drug; PPI, proton pump inhibitor; RCT, randomized controlled trial; Ref., study reference number; USA, United States of America

For the majority of studies, PPI exposure could include any available drug in the class. However, two RCTs administered esomeprazole only [38, 50] and two RCTs administered lansoprazole only [51, 52]. Four studies included exposed participants who were treated with either PPI or H2-receptor antagonist therapy (H2RA). Most studies used pharmacy or prescription records to identify PPI exposure (n = 24, 73%) and diagnostic codes to identify CAP cases (n = 23, 70%).

A total of 6,546,396 study participants were included in our qualitative synthesis. Studies included participants with a breadth of comorbidities which are described in S4 Table; those studies examining CAP within specific disease populations are noted in Table 1. Examples include studies restricting to persons taking anti-Parkinsonian medications [26], to those with newly initiated NSAID therapy [27], to persons with a stroke history [36], or to persons with prior kidney transplantation [40]. total of 262,906 cases of CAP were reported. One study identified CAP cases managed exclusively in the outpatient setting [38]; all other studies presented inpatient or a mix of inpatient and outpatient CAP cases. Individual study definitions of CAP are outlined in S4 Table.

#### Assessment of Methodological Quality

The included observational studies generally demonstrated good methodologic quality, as assessed by a modified Newcastle-Ottawa Scale (S1 Fig) [19]. Case-control studies appropriately conducted and clearly reported exposure and outcome ascertainment. Only one study documented the non-response rate [31]. Cohort studies demonstrated greater risk of bias than case-control studies, particularly with regards to assessment of CAP and follow-up of participants. Among the cohort studies, three showed particularly high risk for bias with respect to our association of interest [34, 40, 44]. The Cochrane Collaboration’s tool [57] was used to assess sequence generation, allocation concealment, and blinding in the included RCTs; all satisfied the requirements for a low risk of bias in these areas. The tool’s assessments of incomplete outcome reporting and selective outcome reporting, however, are not relevant to the studies included in this review because none of these included CAP as a primary outcome. To address the potentially high risk of bias associated with RCTs not designed to assess the association between PPI exposure and CAP, a sensitivity analysis was conducted excluding RCTs from the meta-analysis.

Our funnel plot (S2 Fig) showed clustering of studies below our summary effect estimate. Egger’s test confirmed asymmetry of the funnel plot (0.27; 95% CI 0.14, 0.40; p<0.001). This may reflect a publication bias attenuating our results.

### Meta-Analysis

Our meta-analysis pools data from 26 of the 33 studies included in the systematic review. These 26 studies include 6,351,656 (97.0%) participants and 226,769 (86.2%) cases of CAP. Seven studies were not included in the meta-analysis due to substantially overlapping participant populations [26, 29, 30, 41, 42, 53, 55].

#### Community-Acquired Pneumonia

**Fig 2:**
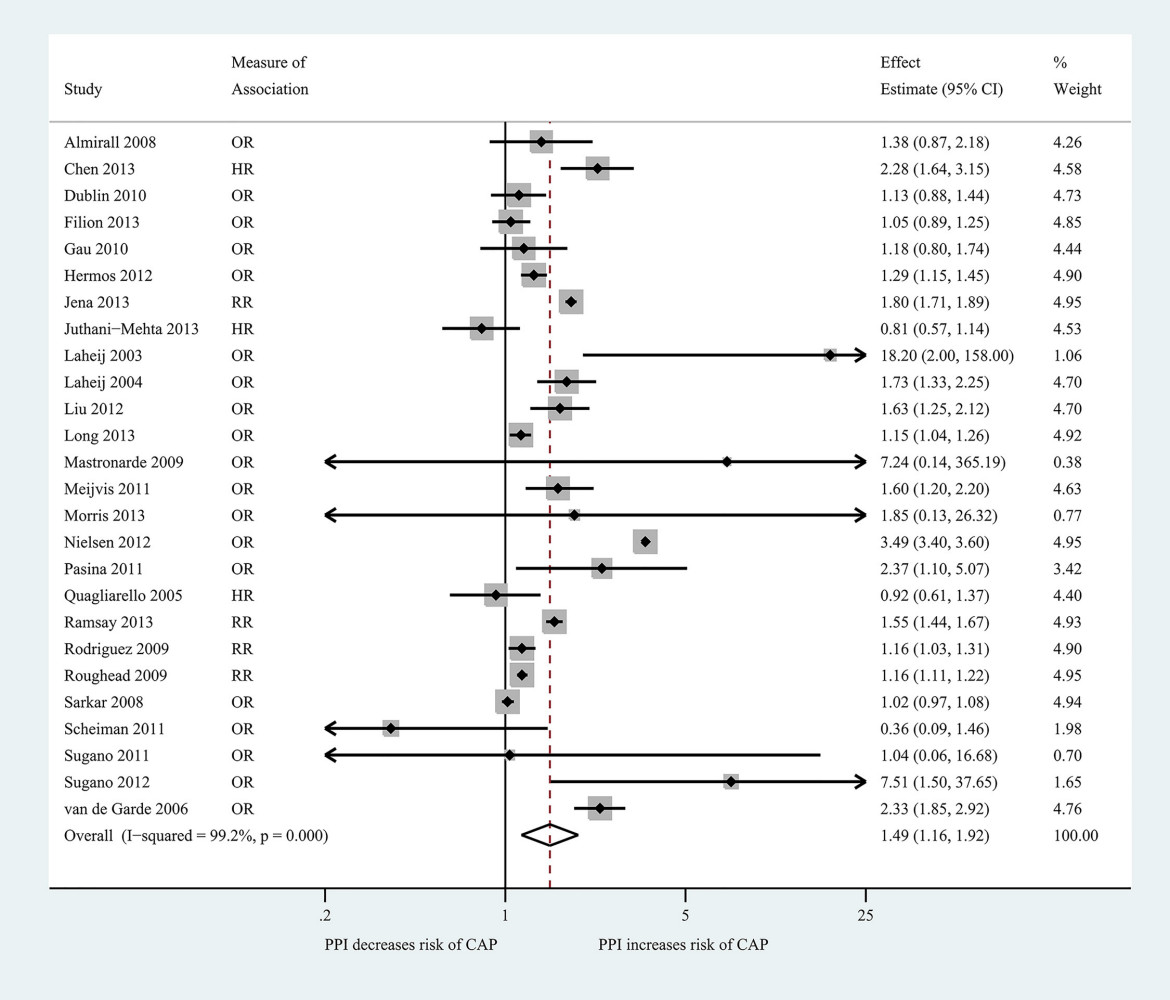
Summary Forest Plot of Overall Risk of Community-Acquired Pneumonia with Outpatient Proton Pump Inhibitor Use, subdivided by study design and effect estimate. Solid diamond represents effect estimate. Shaded box size is proportional to the weight of the study in the meta-analysis. Confidence intervals are denoted by horizontal lines, with arrows where confidence interval extends beyond figure. Vertical dashed line represents the null effect. The open diamond is centered at the summary effect estimate and proportional to the confidence interval.

Of the 26 studies included in our primary meta-analysis, 15 reported a statistically significant increased risk of CAP with PPI use [24, 31, 32, 34–37, 39, 43, 44, 46–48, 52, 54] and 11 reported no statistically significant association [23, 25, 27, 28, 33, 38, 40, 45, 49–51] (Fig 2; S3 Fig). The 15 studies demonstrating significantly increased risk included 138,593 of the total reported 226,769 cases of CAP (61.1%). Four studies reported unadjusted ORs [23, 33, 34, 54]. The Peto method was used to calculate an unadjusted OR for 7 studies [38, 40, 43, 44, 50–52]. Collectively, our meta-analysis identified a significantly increased risk of CAP with outpatient PPI use, with a pooled RR of 1.49 (95% CI 1.16, 1.92). The I^2^ statistic for this summary measure was 99.2% (p<0.001), indicating significant heterogeneity across studies.

#### Sensitivity Analyses

When limiting our analysis to studies that measured PPI exposure separately from other gastric acid suppressant drugs, we found that the association between PPI exposure and CAP remained similar to our primary analysis and heterogeneity remained high (RR 1.48; 95% CI 1.14, 1.92; I^2^ 99.2%) [23–25, 27, 28, 31–33, 35–40, 43, 44, 46–52, 54]. When limiting our analysis to the 6 studies meeting our strict CAP definition which included radiographic confirmation [23, 25, 28, 33, 35, 39, 45], the increased risk of CAP with PPI therapy was attenuated and heterogeneity was reduced (RR 1.22; 95% CI 0.99, 1.52; I ^2^ 65.8%). When we excluded RCTs, point estimates and confidence intervals were nearly identical to those from the primary analysis (RR 1.49; 95% CI 1.15, 1.93; I^2^ 99.3%). Our results were also robust to sensitivity analyses restricting to the 13 studies identified as having lower risk of bias and to studies reporting adjusted measures of association (S5 Table).

### Secondary Analyses

#### Risk of Hospitalization for CAP with PPI Therapy

We pooled 16 studies in order to evaluate the secondary outcome of hospital admission for CAP among PPI users compared to non-PPI users [23, 24, 27, 28, 33, 36, 39, 43, 46–52, 54]. The pooled relative risk of hospitalization with CAP was 1.61-fold higher among PPI users as compared to non-PPI users (95% CI: 1.12, 2.31; I^2^ 99.3%).

#### Risk of CAP with H2RA Therapy

In order to evaluate CAP risk with H2RA therapy, we pooled 8 studies that reported this risk separately from the risk associated with PPI therapy [23, 25, 27, 28, 35, 47, 49, 51]. Combined, these studies included 4837 cases of CAP. When pooled, these studies demonstrated no significant CAP risk with H2RA therapy as compared to no acid-suppression therapy (RR 1.00; 95% CI 0.90, 1.12; I^2^ 33.7%).

### Subgroup Analyses

Subgroup analyses are summarized in Table 2. The 8 studies reporting risk of CAP with low dose PPI therapy (≤ 1 DDD), as compared to no PPI therapy [23, 28, 39, 47, 49–52], identified a pooled RR of 1.31 (95% CI 1.04, 1.66), which was similar to findings among those taking high dose PPI therapy (RR 1.33; 95% CI 1.05, 1.69) [23, 28, 35, 39, 47, 49, 50]. Treatment with a PPI for less than 1 month was associated with the highest risk of CAP (OR 2.10; 95% CI 1.39, 3.16), as compared to no PPI therapy [23, 35, 39, 47, 49, 51]. The magnitude of CAP risk decreased and lost statistical significance as duration of PPI therapy increased (OR 1.51; 95% CI 0.92, 2.49 for 1–6 months [23, 35, 49, 51, 52] and OR 1.37; 95% CI 0.85, 2.20 for >6months) [23, 35, 49, 51, 52]. Participants aged ≥ 65 years demonstrated significantly increased risk of CAP with PPI therapy as compared to those not taking PPIs (OR 1.33; 95% CI 1.13, 1.58; I^2^ 85.4%) [23–25, 28, 31, 45, 46, 48, 50–52]. Similar findings were observed among participants <65 years old (OR 1.34; 95% CI 1.04, 1.71; I^2^ 60.1%) [23, 24, 31, 37, 50–52].

**Table 2:** Summary of Subgroup Analyses.

Subgroup		# of Studies	Pooled Effect Estimate	95% CI	I^2^ (%)	p-value for Heterogeneity	Reference
PPI Dose*	High	7	1.33	1.05–1.69	34.0	0.168	23,28,35,39,47,49,50
	Low	8	1.31	1.04–1.66	71.4	0.001	23,28,39,47,49,50,51,52
PPI Duration†	<1 month	6	2.10	1.39–3.16	72.5	0.003	23,35,39,47,49,51
	1–6 months	5	1.51	0.92–2.49	63.3	0.028	23,35,49,51,52
	>6 months	5	1.37	0.85–2.20	74.1	0.004	23,35,49,51,52
Age	<65 years	7	1.34	1.04–1.71	60.1	0.020	23,24,31,37,50,51,52
	>65 years	11	1.33	1.13–1.58	85.4	<0.001	23,24,25,28,31,45,46,48,50,51,52

* Dose is categorized as Low for doses ≤ 1 defined daily dose, High for doses > 1 defined daily dose

† Duration refers to duration of time taking PPI prior to community-acquired pneumonia diagnosis

## Discussion

This systematic review of 33 studies and meta-analysis of 26 studies demonstrated a 1.5-fold increased risk of community-acquired pneumonia with outpatient proton-pump inhibitor therapy. PPI therapy was also associated with an increased risk for hospitalization with CAP (1.6-fold). No association was observed between H2RA use and CAP among studies examining participants taking H2RA therapy alone. CAP risk did not vary by PPI dose or participant age but more than doubled among those treated for less than 1 month, as compared to those not taking PPIs. Our findings were robust to sensitivity analyses restricting to studies exploring only PPI therapy (excluding H2RA therapy), studies utilizing a CAP definition requiring radiographic confirmation, and studies found to have lower risk of bias during qualitative analysis.

Several pathogenic mechanisms have been proposed to explain the association between PPI use and incidence of CAP. Decreased gastric acidity is associated with alteration of gut flora [58–61]. Micro-aspiration of the altered gut flora is one hypothesized mechanism for the increased CAP risk observed in the setting of elevated gut pH. More recently, proton pumps have been localized to the upper and lower respiratory tract [62, 63], suggesting that pH dysregulation may additionally alter respiratory flora, thereby directly inducing infection [64]. Our findings are consistent with the theory that PPI therapy may lead to CAP both through acute pH dysregulation and alteration of the gut microbiome. CAP risk was greatest during the first month of therapy, which is the time period during which the aero-digestive microbiome may be in greatest flux [65]. This association between CAP risk and short duration of PPI therapy, in conjunction with a lack of dose and duration effect, suggest that the acute changes occurring with onset of PPI therapy may be responsible for CAP risk. Our findings cannot define a causal relationship but do highlight an important association that requires further investigation. CAP risk was absent among participants taking H2RAs, which have weaker acid suppressant activity than do PPIs, however our study was not constructed to fully investigate this question.

Given the widespread usage of PPI therapy, often without an appropriate indication, the excess risk of CAP among PPI users could translate into a substantial burden on the healthcare system. Moreover, the increased risk of hospitalization for CAP underscores the potential clinical and financial impact of this adverse effect. Careful consideration of the risks, benefits and alternative treatment options should occur with all PPI prescriptions. Our observation that CAP risk was increased with PPI therapy, regardless of PPI dose or participant age, implies that alternate therapies, when appropriate, may be a strategy for reducing CAP risk. Secondary analysis showing an absence of CAP risk among participants taking H2RAs further supports the reduced risk profile of alternate therapies.

Our systematic review and meta-analysis could be subject to several sources of bias. Confounding by indication may have augmented our findings if the incidence of CAP is higher among persons taking PPIs due to the nature of their disease state, unrelated to the PPI therapy. GERD, a common indication for PPI therapy [12, 65], may directly increase risk of CAP by increasing microaspiration of gut flora. Several studies included in our review utilized different methods to reduce this source of confounding. Four studies restricted study enrollment to participants with a non-GERD indication for PPI [27, 40, 51, 52], one study employed a case-crossover design so that participants in both the exposed and unexposed groups possessed the same comorbidities [36], and two studies compared current PPI users to former PPI users to limit variability in comorbidities between cases and controls [31, 35]. Jena, *et al*., further explored the concept of confounding by using prescription drug and medical claims data to demonstrate statistically significant associations between PPI use and conditions without a readily apparent pathophysiologic link to PPI use, such as osteoarthritis, chest pain, and urinary tract infection. The authors raise the important point that the observed relationship between PPI use and CAP may be influenced by unmeasured confounders. This warrants further investigation, including replication of the findings using clinically-driven criteria for disease outcomes and efforts to elucidate specific confounders that may be driving any associations. Importantly, the absence of a known pathophysiologic mechanism for a causal relationship does not preclude the existence of one.

Reporting bias is an additional source of bias inherent to systematic reviews and meta-analyses. Our search was designed to detect a broad array of study designs respiratory diagnoses, yet only identified four RCTs reporting pneumonia as an adverse event. The four RCTs included in our review contribute few patients to the primary analysis, were not designed specifically to address our study question, and relied upon adverse event reporting to assess the incidence of pneumonia. Still, our findings were essentially unchanged in multiple sensitivity analyses, including an analysis that excluded RCTs.

Publishing bias may have also influenced our findings. Though our funnel plot was limited by the number of included studies [56, 66], it suggested a publication bias toward a protective or null effect of PPI therapy. If present, the magnitude of the association we report may actually be smaller than the true effect.

Our study has limitations. Several studies reported data on overlapping participant populations. We selected only the most inclusive studies but cannot guarantee inclusion of all eligible participants in our meta-analysis. The included studies reported one of three different measures of association: odds ratio, hazard ratio, or relative risk. Because CAP is a rare outcome, the odds ratio and hazard ratio are each assumed to approximate the relative risk. We observed the greatest risk for CAP in the setting of PPI use for less than 1 month, which may have a biological explanation but may also, at least partly, reflect a protopathic bias due to initiation of PPI therapy around the time of the development of respiratory symptoms.

Tests for heterogeneity demonstrated variability of findings across studies; however consistent demonstration of increased CAP risk with PPI therapy and the findings from our qualitative analysis suggested that pooling of the 26 identified studies for meta-analysis was reasonable despite differences in methodology. The impact of these differences was assessed in subgroup, secondary, and sensitivity analyses. In many cases, studies demonstrated significantly less heterogeneity when grouped for these additional analyses. The subgroup analysis of the association between high-dose PPI exposure and CAP demonstrated the least heterogeneity (I^2^ 34.0%). Almost all of these analyses supported our primary findings. Among sensitivity analyses, only the analysis examining the strictest CAP definition failed to show a significant association between PPI exposure and CAP. With only 7 included studies, representing 3,865 CAP cases among 16,537 participants, this sensitivity analysis was likely underpowered to detect a significant difference.

Several previous meta-analyses have sought to evaluate the relationship between PPI therapy and CAP. Giuliano, et al, concluded that patients prescribed PPIs had an increased risk for CAP, particularly at high PPI doses and soon after initiation of PPI therapy [67]. Eom, et al., found that patients prescribed any acid suppressant therapy were at increased risk of community-acquired or hospital-acquired pneumonia [68]. Our meta-analysis enhances the existing literature by adding data from 19 studies that were not included in these prior investigations. These 19 studies were identified through a combination of our broad, updated database search and our extensive pursuit of unpublished data. The inclusion of unpublished data also strengthened our subgroup and sensitivity analyses.

In conclusion, we conducted a thorough systematic review and rigorous meta-analysis of studies reporting the risk of CAP with ambulatory PPI therapy. We identified a 1.5-fold increased risk for CAP with outpatient PPI therapy, with additional analyses revealing even greater risk during the first month of therapy and for CAP hospitalization. Our findings add to currently available literature through the breadth of our search, inclusion of unpublished data and updated research, and extent of analysis. Given the morbidity and mortality associated with CAP and the extent of PPI use in the United States, identification of any risk associated with PPI use is critical for risk stratification and modification where possible. Future studies should employ a rigorous definition of CAP and ascertain PPI exposure at various doses and durations to further explore the association between PPI exposure and CAP within specific populations. Studies are also needed to clarify any pathophysiologic mechanisms underlying the observed association. We recommend careful consideration of the risks and benefits when initiating PPI therapy and heightened awareness regarding this risk factor for adults presenting with pneumonia.

## Supporting Information

S1 Dataset (DTA)

S1 Fig Risk of Bias Assessments for Observational Studies. Risk of bias was assessed using a modified Newcastle-Ottawa Scale as outlined in S3 Table. The risk associated with each criterion on the scale corresponds to a circle in this figure. Low risk is represented by a green circle, medium risk yellow, and high risk red.
(PDF)

S2 Fig Funnel plot for Primary Outcome with Pseudo-95% Confidence Limits. This funnel plot displays standard error of the effect estimate as a measure of study size on the vertical axis and estimated effect of PPI therapy on CAP diagnosis on the horizontal axis. Dashed lines represent pseudo-95% confidence interval lines, drawn around the summary fixed-effect estimate of the effect of PPI therapy on CAP diagnosis.
(PDF)

S3 Fig Forest Plot for Primary Analysis, Stratified by Study Design. (PDF)

S1 Table PRISMA 2009 Checklist. (PDF)

S2 Table Representative Search String for PubMed. We performed systematic searches of MEDLINE (via PubMed), EMBASE, CINAHL, Cochrane Central Register of Controlled Trials (CENTRAL), Scopus, Web of Science and ClinicalTrials.gov on February 3, 2014. All search strings included controlled vocabulary and related keywords for two concepts: pneumonia and acid suppressants. A representative search string used for MEDLINE (via PubMed) is presented here. Concept terms were combined via the Boolean operator “AND” for database searches.
(PDF)

S3 Table Modified Newcastle-Ottawa Scale for Risk of Bias Assessment. (PDF)

S4 Table Patient and Study Characteristics of the 33 Studies under Systematic Review (PDF)

S5 Table Summary of Sensitivity Analyses. (PDF)
