# Supplementary material for: Risk of Community-Acquired Pneumonia with Outpatient Proton-Pump Inhibitor Therapy: A Systematic Review and Meta-Analysis
Source: PLoS One. 2015 Jun 4;10(6):e0128004. doi: 10.1371/journal.pone.0128004 (PMC4456166; doi:10.1371/journal.pone.0128004)
Supplement: S1 Fig — Risk of bias was assessed using a modified Newcastle-Ottawa Scale as outlined in S3 Table. The risk associated with each criterion on the scale corresponds to a circle in this figure. Low risk is represented by a green circle, medium risk yellow, and high risk red. (PDF) [file pone.0128004.s002.pdf]

S5 Figure. Risk of Bias Assessments for Observational Studies

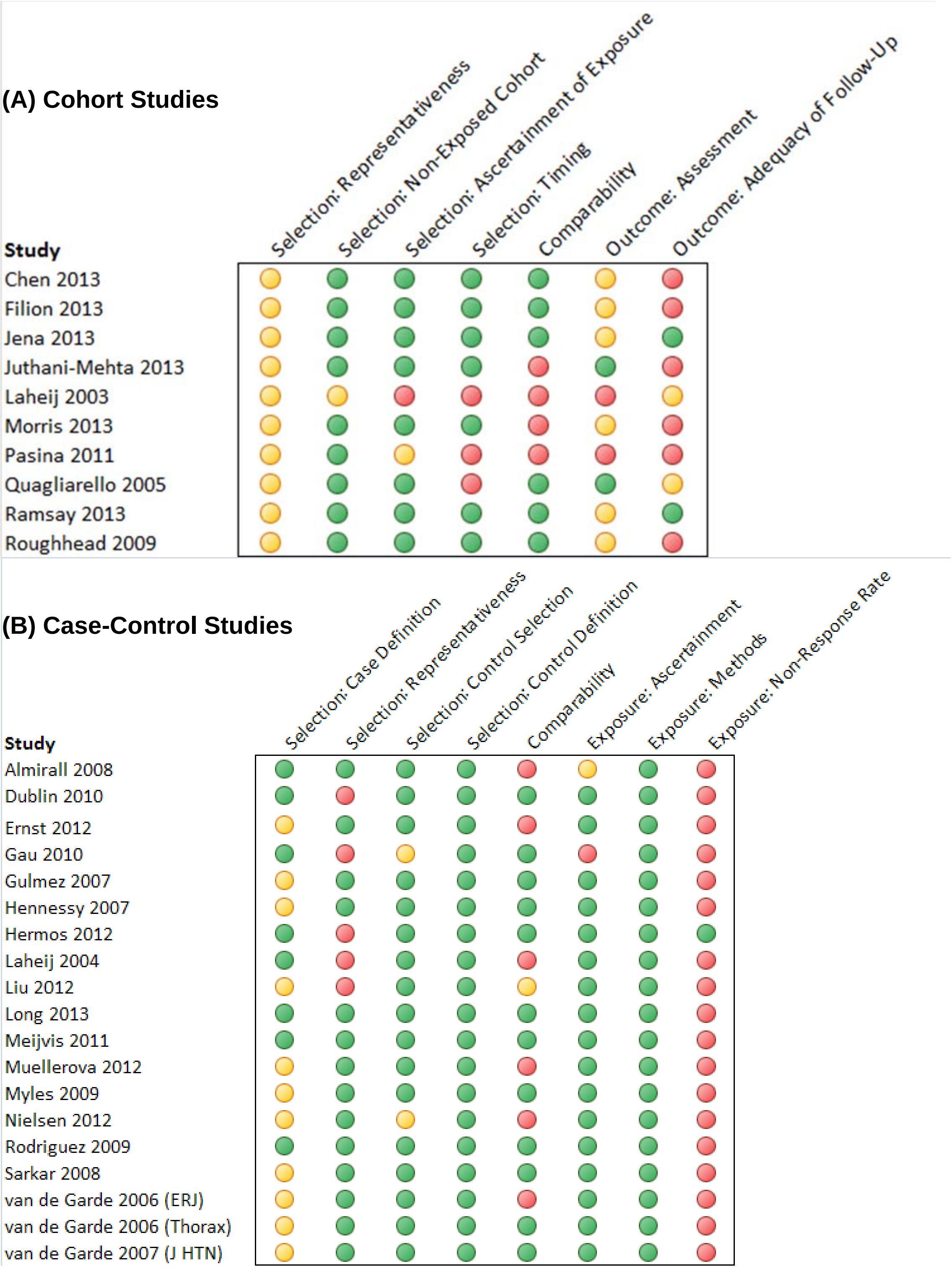

Risk of bias was assessed using a modified Newcastle-Ottawa Scale as outlined in Online Supplemental Table 3. The risk associated with each criterion on the scale corresponds to a circle in this figure. Low risk is represented by a green circle, medium risk yellow, and high risk red.
