# Supplementary material for: Risk of Community-Acquired Pneumonia with Outpatient Proton-Pump Inhibitor Therapy: A Systematic Review and Meta-Analysis
Source: PLoS One. 2015 Jun 4;10(6):e0128004. doi: 10.1371/journal.pone.0128004 (PMC4456166; doi:10.1371/journal.pone.0128004)
Supplement: S2 Fig — This funnel plot displays standard error of the effect estimate as a measure of study size on the vertical axis and estimated effect of PPI therapy on CAP diagnosis on the horizontal axis. Dashed lines represent pseudo-95% confidence interval lines, drawn around the summary fixed-effect estimate of the effect of PPI therapy on CAP diagnosis. (PDF) [file pone.0128004.s003.pdf]

S6 Figure. Funnel plot for Primary Outcome with Pseudo-95% Confidence Limits

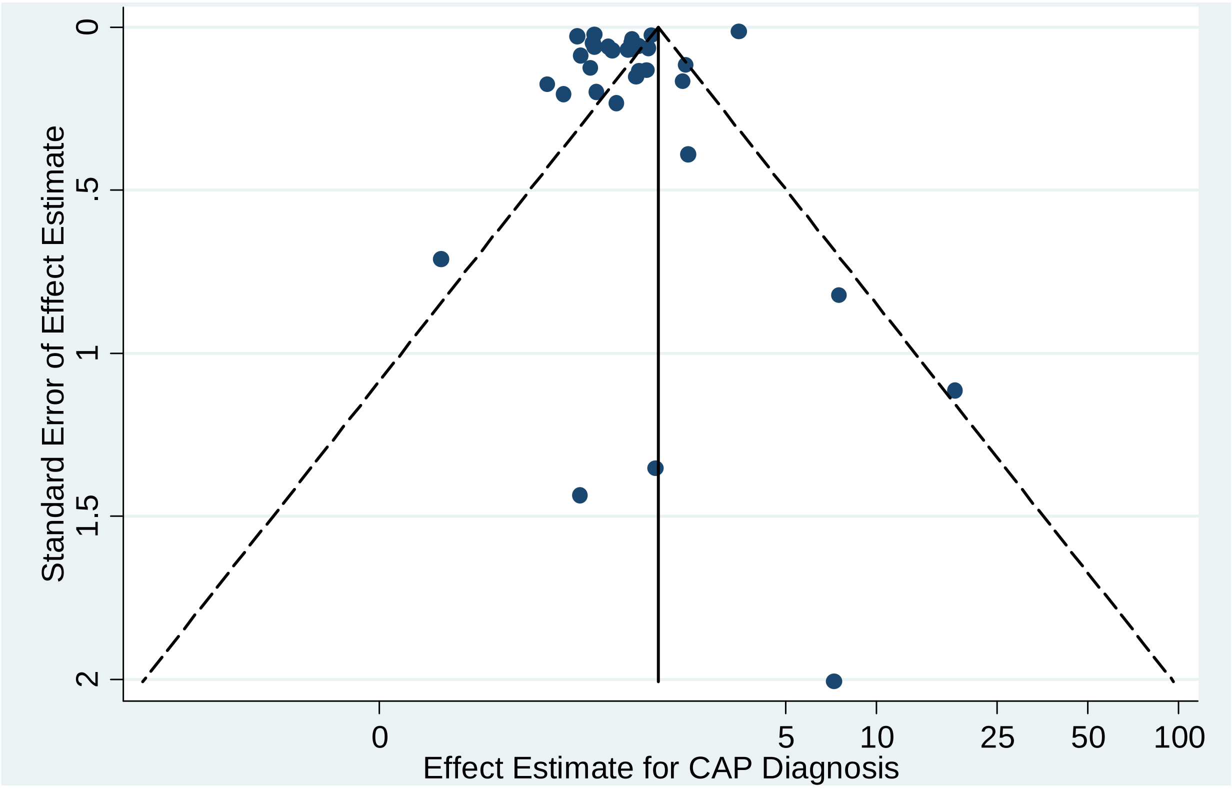

This funnel plot displays standard error of the effect estimate as a measure of study size on the vertical axis and estimated effect of PPI therapy on CAP diagnosis on the horizontal axis. Dashed lines represent pseudo-95% confidence interval lines, drawn around the summary fixed-effect estimate of the effect of PPI therapy on CAP diagnosis.
