# Supplementary figures and images for: Risk of Community-Acquired Pneumonia with Outpatient Proton-Pump Inhibitor Therapy: A Systematic Review and Meta-Analysis
Source: PLoS One. 2015 Jun 4;10(6):e0128004. doi: 10.1371/journal.pone.0128004 (PMC4456166; doi:10.1371/journal.pone.0128004)

**S7 Figure. Forest Plot for Primary Analysis, Stratified by Study Design.**

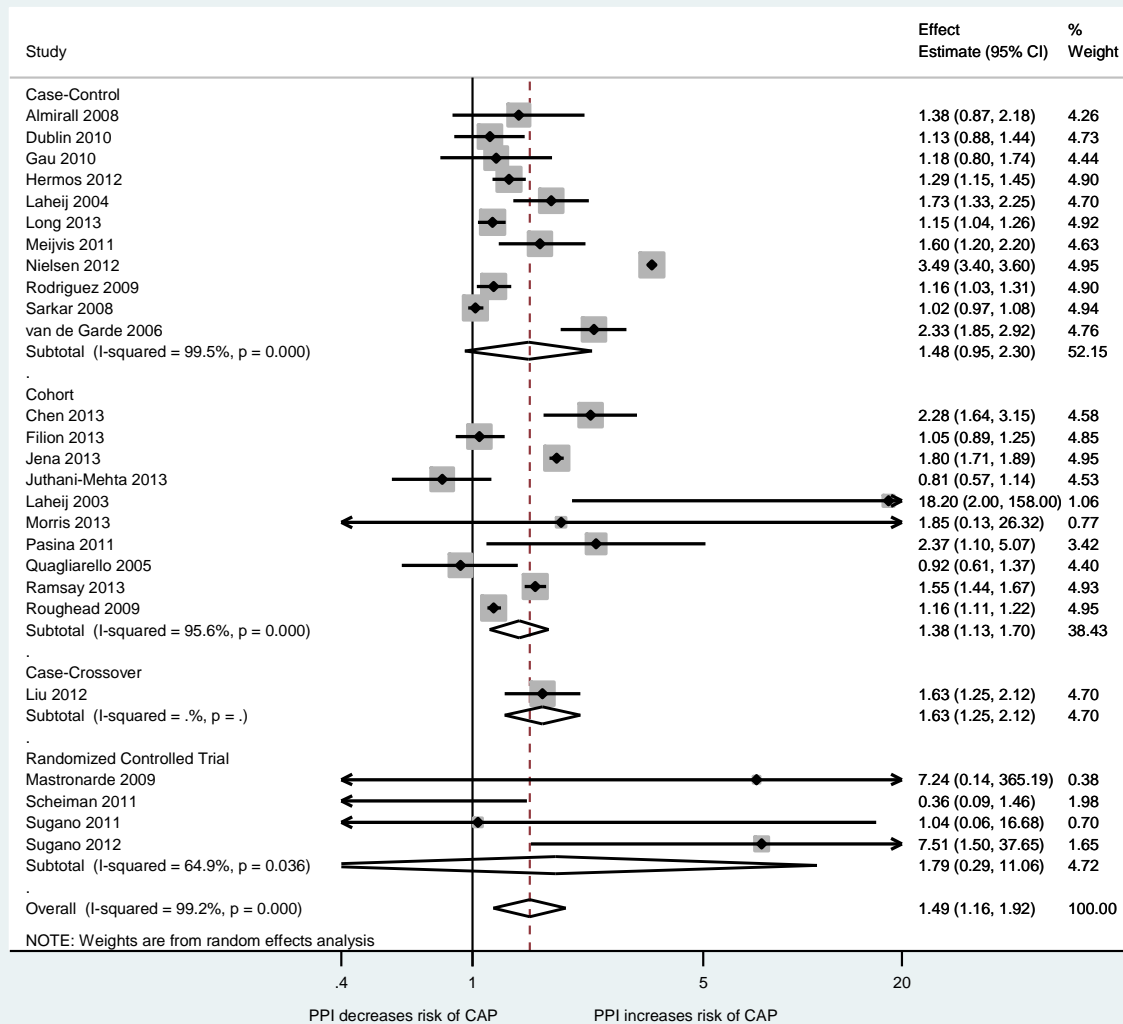

Supplement: S3 Fig — (PDF) [file pone.0128004.s004.pdf]
