# Supplementary material for: Risk of Community-Acquired Pneumonia with Outpatient Proton-Pump Inhibitor Therapy: A Systematic Review and Meta-Analysis
Source: PLoS One. 2015 Jun 4;10(6):e0128004. doi: 10.1371/journal.pone.0128004 (PMC4456166; doi:10.1371/journal.pone.0128004)
Supplement: S2 Table — We performed systematic searches of MEDLINE (via PubMed), EMBASE, CINAHL, Cochrane Central Register of Controlled Trials (CENTRAL), Scopus, Web of Science and ClinicalTrials.gov on February 3, 2014. All search strings included controlled vocabulary and related keywords for two concepts: pneumonia and acid suppressants. A representative search string used for MEDLINE (via PubMed) is presented here. Concept terms were combined via the Boolean operator “AND” for database searches. (PDF) [file pone.0128004.s006.pdf]

S2 Table. Representative Search String for PubMed

Concept	Search String
Acid Suppressants	(Proton pump inhibitors[MeSH Terms] OR "Proton Pumps/antagonists and inhibitors"[MeSH Terms] OR "Proton pump inhibitor"[tw] OR "Proton pump inhibitors"[tw] OR PPI[tw] OR "Esomeprazole Sodium"[MeSH Terms] OR Esomeprazole[tw] OR Lansoprazole[tw] OR omeprazole[MeSH Terms] OR Omeprazole[tw] OR Pantoprazole[tw] OR Rabeprazole[tw] OR Dexlansoprazole[tw] OR PPIs[tw] OR "H(+)-K(+)-Exchanging ATPase"[MeSH Terms] OR Ilaprazole[tw] OR benatoprazole[tw] OR "H(+)-K(+)-Exchanging ATPase"[tw] OR "hydrogen potassium atpase inhibitor"[tw] OR "hydrogen potassium atpase inhibitors"[tw] OR leminoprazole[tw] OR picoprazole[tw] OR pumaprazole[tw] OR timoprazole[tw] OR "prilosec"[tw] OR nexium[tw] OR "potassium competitive acid blocker"[tw] OR "potassium competitive acid blockers"[tw] OR anti-ulcer agents[MeSH Terms] OR "anti ulcer agents"[tw] OR "anti-ulcer agents"[tw] OR "anti-ulcer drug"[tw] OR "antiulcer drug"[tw] OR antacid[tw] OR antacids[MeSH Terms] OR "antisecretory agents"[tw] OR "antisecretory agent"[tw] OR "stomach secretion inhibition"[tw] OR "gastric secretion inhibitor"[tw] OR "antiulcer drugs"[tw] OR "gastric secretion inhibition"[tw] OR "gastric secretion inhibitors"[tw] OR "hydrogen potassium adenosine triphosphatase inhibitor"[tw] OR "hydrogen potassium adenosine triphosphatase inhibitors"[tw] OR "antisecretory agents"[tw] OR "Stomach secretion inhibitor"[tw] OR "Stomach secretion inhibitors"[tw] OR "anti-ulcer drugs"[tw] OR "gastric acid suppressor"[tw] OR "gastric acid suppressors"[tw] OR "gastric acid inhibitor"[tw] OR "gastric acid inhibitors"[tw] OR "gastric acid suppression"[tw] OR "gastric acid inhibition"[tw] OR "stomach secretion inhibiting"[tw] OR "gastric acid suppressing"[tw] OR "gastric acid inhibiting"[tw] OR "stomach secretion suppressing"[tw] OR "gastrointestinal mucosa protective agent"[tw] OR "gastrointestinal mucosa protective agents"[tw] OR "gastrointestinal mucosa protecting"[tw] OR "gastrointestinal mucosa protection"[tw] OR revaprazan[tw] OR saviprazole[tw] OR oraprazan[tw] OR soraprazan[tw] OR vonoprazan[tw] OR "anti-ulcer agent"[tw] OR "anti-ulcer drugs"[tw] OR "antiulcer drugs"[tw] OR "antiulcer agent"[tw] OR "antiulcer agents"[tw] OR antacids[tw] OR "rabeprazole" [Supplementary Concept] OR "timoprazole"[Supplementary concept] OR "lansoprazole"[Supplementary Concept] OR "pantoprazole"[Supplementary Concept] OR "anti-ulcer agents" [pharmacological action] OR "antacid"[pharmacological action] OR "proton pump antagonist" OR "proton pump antagonists")
Pneumonia	(Pneumonias[tw] OR Pneumonia[tw] OR CAP[tw] OR Pneumonia[tw] OR Community-acquired pneumonia[tw] OR Peripneumonia[tw] OR Pneumonitides[tw] OR Lobitis[tw] OR "Lobe infiltrate"[tw] OR "Lobar infiltrate"[tw] OR Bronchopneumonia[tw] OR pleuropneumonia[tw] OR pleuropneumonias[tw] OR Pleuritis[tw] OR Community acquired infections[MeSH Terms] OR "Community acquired infections"[tw] OR "Community acquired infection"[tw] OR PNA[tw] OR PNAs[tw] OR CAPs[tw] OR pneumonic lung[tw] OR pneumonitis[tw] OR "pulmonary inflammation"[tw] OR "community acquired"[tw] OR "community-acquired"[tw] OR "pulmonic inflammation"[tw] OR "pulmonary inflammations"[tw] OR "pulmonic inflammations"[tw] OR Pneumonia[MeSH Terms] OR "lung infection"[tw] OR "lung infections"[tw] OR "Respiratory infection"[tw] OR "Respiratory infections"[tw] OR "Respiratory tract infection"[tw] OR "Respiratory tract infections"[tw] OR "Pulmonary infection"[tw] OR "Pulmonary infections"[tw] OR "Chest infection"[tw] OR "Chest infections"[tw] OR bronchitis[tw] OR Tracheobronchitis[tw] OR Laryngobronchitis[tw] OR "Respiratory tract inflammation"[tw] OR bronchopneumonias[tw] OR Laryngotracheobronchitis[tw])

We performed systematic searches of MEDLINE (via PubMed), EMBASE, CINAHL, Cochrane Central Register of Controlled Trials (CENTRAL), Scopus, Web of Science and ClinicalTrials.gov on February 3, 2014. All search strings included controlled vocabulary and related keywords for two concepts: pneumonia and acid suppressants. A representative search string used for MEDLINE (via PubMed) is presented here. Concept terms were combined via the Boolean operator “AND” for database searches.
