# Supplementary material for: Risk of Community-Acquired Pneumonia with Outpatient Proton-Pump Inhibitor Therapy: A Systematic Review and Meta-Analysis
Source: PLoS One. 2015 Jun 4;10(6):e0128004. doi: 10.1371/journal.pone.0128004 (PMC4456166; doi:10.1371/journal.pone.0128004)
Supplement: S3 Table — (PDF) [file pone.0128004.s007.pdf]

S3 Table. Modified Newcastle-Ottawa Scale for Risk of Bias Assessment

Case Control Studies	Cohort Studies
**Selection**<br>1) Is the case definition adequate?<br>a) yes, with independent validation<br>b) yes, e.g., record linkage or based on self-reports<br>c) no description<br>2) Representativeness of the cases<br>a) consecutive or obviously representative series of cases<br>b) potential for selection biases or not stated<br>3) Selection of Controls<br>a) community controls<br>b) hospital controls<br>c) no description<br>4) Definition of Controls<br>a) no history of disease (endpoint)<br>b) no description of source	**Selection**<br>1) Representativeness of the exposed cohort<br>a) truly representative of the average adult in the community<br>b) somewhat representative of the average adult in the community<br>c) selected group of users e.g. ,nurses, volunteers<br>d) no description of the derivation of the cohort<br>2) Selection of the non-exposed cohort<br>a) drawn from the same community as the exposed cohort<br>b) drawn from a different source<br>c) no description of the derivation of the non-exposed cohort<br>3) Ascertainment of exposure<br>a) secure record (e.g., surgical records)<br>b) structured interview<br>c) written self-report<br>d) no description<br>4) Demonstration that outcome of interest was not present at study start<br>a) yes<br>b) no
**Comparability**<br>1) Comparability of cases and controls on the basis of the design or analysis<br>a) study controls for age<br>b) study controls for any additional factor	**Comparability**<br>1) Comparability of cohorts on the basis of the design or analysis<br>a) study controls for age<br>b) study controls for any additional factor
**Exposure**<br>1) Ascertainment of exposure<br>a) secure record (e.g., pharmacy records)<br>b) structured interview where blind to case/control status<br>c) interview not blinded to case/control status<br>d) written self-report or medical record only<br>e) no description<br>2) Same method of ascertainment for cases and controls<br>a) yes<br>b) no<br>3) Non-Response rate<br>a) same rate for both groups<br>b) non respondents described<br>c) rate different and no designation	**Outcome**<br>1) Assessment of outcome<br>a) independent blind assessment<br>b) record linkage<br>c) self-report<br>d) no description<br>2) Adequacy of follow up of cohorts<br>a) complete follow up - all subjects accounted for<br>b) subjects lost to follow up unlikely to introduce bias: i.e.,<10% small number lost, or description provided of those lost<br>c) follow-up rate < 90% and no description of those lost<br>d) no statement
