# Supplementary material for: Risk of Community-Acquired Pneumonia with Outpatient Proton-Pump Inhibitor Therapy: A Systematic Review and Meta-Analysis
Source: PLoS One. 2015 Jun 4;10(6):e0128004. doi: 10.1371/journal.pone.0128004 (PMC4456166; doi:10.1371/journal.pone.0128004)
Supplement: S4 Table — (PDF) [file pone.0128004.s008.pdf]

S4 Table. Patient and Study Characteristics of the 33 Studies under Systematic Review

Author Year	Age, years	CAP Definition	Comorbidities	Comparison	Additional Covariates	Ref.
Almirall 2008	Male cases: 58.6 (19.8)<br>Female cases: 54.6 (20.7)<br>Male controls: 58.9 (19.6)<br>Female controls 54.6 (20.6)	Medical record review with prescription of antibiotics and new radiological findings suggestive of infiltrate	Asthma, CAD, CHF, COPD, CVA, Dementia, Dental dysaethesia, Dental prosthesis, DM, Epilepsy, GERD, Heart valve disease, HIV, Liver disease, Nonactive pulmonary tuberculosis, Parkinson's disease, Psychiatric disorders, Renal failure, Visit to dentist in last month	Matched 1:1 on age, sex, and primary care center using frequency matching to patients in same primary care center	None	23
Chen 2013	Exposed: 63.3 (13.0)<br>Unexposed: 54.2 (18.3)	Diagnostic codes (ICD-9)	Acute renal failure*, asthma*, CKD, COPD*, CVA*, DM*, ischemic heart disease*	Unexposed cohort members	Age, sex	24
Dublin 2010	Median: 77 (IQR: 71-82)	Diagnostic codes (ICD-9), confirmed by review of radiology and hospital records	Alcoholism*, Asthma*, CHF*, COPD*, Coronary revascularization*, CVA*, Dementia*, DM, Dyspepsia, Frailty*, Functional impairment, GERD, MI*, Need for home oxygen*, Peptic ulcer, Swallowing disorder leading to aspiration*, Tobacco use*	Matched 1:2 on age, sex, and calendar year using incidence density sampling	COPD hospitalizations, FEV1, CHF hospitalization, ejection fraction, other heart disease, pneumococcal or influenza vaccination, number of outpatient visits, need for assistance with ambulation or bathing<br>Medications: inhaled bronchodilators, inhaled corticosteroids, oral corticosteroids, furosemide, H2RA	25
Ernst 2012	Cases: 79.2 (8.0)<br>Controls: 78.6 (7.8)	Diagnostic codes (ICD-10)	Asthma, Anemia, Bipolar disorder, CAD, Cancer, Cardiac valve condition, CHF, COPD, CVA, Dementia, Depression, Epilepsy, Hypertension, Motor neuron disease, Peripheral edema, Psychosis, Renal failure, Rhythm irregularity	Matched 1:up to 10 on age, indication for anti-parkinsonian drug, prevalent or new user at cohort entry, and year of cohort entry using patients in same database	None	26

Author Year	Age, years	CAP Definition	Comorbidities	Comparison	Additional Covariates	Ref.
Filion 2013	Exposed: 65-74 (30.5%)<br>Unexposed: 40-54 (41.6%)	Diagnostic codes (ICD-10)	Asthma*, COPD*, Bronchiectasis*, DM*, Excessive alcohol use*. Tobacco use*	Unexposed cohort members	index year, income category, hospitalization in previous year, use of >4 medication in previous year, >4 physician visits in the previous year, BMI, up to 500 additional confounders empirically included in propensity score<br>Medications: immunosuppressive agents, inhaled bronchodilators, inhaled corticosteroids, non-topical antibiotics, non-topical corticosteroids, influenza or pneumococcal vaccines.	27
Gau 2010	Cases: 80.3 (8.5)<br>Controls: 79.8 (8.1)	Discharge diagnosis with radiographic confirmation	Atrial fibrillation, CAD*, CHF*, Cognitive impairment, COPD*, CVA, Depression, DM, GERD, Prior pneumonia, Psychiatric illness	Hospitalized controls without pneumonia or COPD exacerbation	Age, sex, smoking status, serum albumin<br>Medications: atypical antipsychotics, beta2-agonists, anti-cholinergics, inhaled corticosteroids, iron, narcotics, NSAIDs, antibiotics	28
Gulmez 2007	Cases: 55.5 (31.2)<br>Controls: 56.5 (29.5)	Diagnostic codes (ICD-8 & ICD-10)	COPD*, Peptic ulcer*, Heart failure*, Ischemic heart disease*, DM*, CVA*, Renal failure*, Hepatic cirrhosis*, Alcohol-related* diagnosis, drug use diagnosis, psychiatric disorder	Matched 1:4 on age in 10-year band, and sex using randomly selected community controls	Previous discharge diagnosis of CAP, ischemic heart disease, liver cirrhosis, renal failure<br>Medications: systemic and inhaled corticosteroids, bronchodilators, NSAIDs, anticholinergic agents, antipsychotic agents	29
Hennessy 2007	Cases: 81 (74-86)<br>Controls: 75 (70-81)	Diagnostic codes (Read)	Alcohol use, Anterior horn motor neuron disease, Anxiety, Bipolar disorder, COPD, CVA, Depression, Dysphagia, Herpetic neuralgia/herpes zoster, Insomnia, Parkinson's Disease, Poor nutritional status, Schizophrenic disorders, Senile dementia, Suicidal ideation, Tobacco use	Matched1:4 on general practitioner practice group using incidence density sampling of patients in same database	Age, sex, calendar year<br>Medications: current use of antidepressant drug	30

Author Year	Age, years	CAP Definition	Comorbidities	Comparison	Additional Covariates	Ref.
Hermos 2012	65.8 (12.2)	Diagnostic code (ICD-9) and pharmacy record of respiratory antibiotic prescription	Alcohol/drug dependence or abuse*, CHF*, Chronic liver disease*, CKD*, COPD*, Dementia*, DM*, GERD*, H. pylori, Ischemic heart disease*, Lung cancer*, Non-skin/non-lung cancer*, Peptic ulcer diseases*, Reflux esophagitis*	Matched 1:10 on age, and duration under observation using risk set sampling of patients in same database. Controls were former PPI users.	Sex, time period of CAP diagnosis <u>Medications</u>: H2RA, systemic corticosteroid, immunomodulator/immunosuppressant, tranquilizer/sedative, or antipsychotic agents active use at baseline; antibiotic use ≤90 days before baseline.	31
Jena 2013	Exposed: 66.5 (0.1) Unexposed: 66.0 (0.1)	Diagnostic codes (ICD-9)	Asthma/COPD*, cancer*, chest pain, chronic renal insufficiency*, congestive heart failure*, coronary artery disease*, deep vein thrombosis, diabetes*, hyperlipidemia*, hypertension*, osteoarthritis, rheumatoid arthritis, skin infection, stroke*, urinary tract infection	Controls never exposed to PPI in the same insurance claims database	Age, geographic location, income, marital status	32
Juthani-Mehta 2013	Cases: 75.2 (2.8) Controls: 74.7 (2.9)	Medical record review of radiography, respiratory symptoms, physical examination, and diagnostic codes (ICD-9)	Cancer, CAD, chronic lung disease, CVA, depression, DM, hypertension, tobacco use	Unexposed cohort members	None	33
Laheij 2003	Exposed: >60 (49%) Unexposed: >60 (37%)	Patient report	Asthma/COPD, Current tobacco or alcohol use, Foreign country visit, Obesity	Unexposed cohort members	None	34
Laheij 2004	Cases: >60 (58.1%) Controls: >60 (58.1%)	Medical record review of radiography, microbiology or respiratory symptoms	COPD, DM*, Heart failure*, Indication for antacids (GERD, cancer, peptic ulcer disease, functional dyspepsia), Lung cancer, Stomach cancer	Matched 1:10 on sex, birth year, and index date using Integrated Primary Care Information project enrollees	Respiratory illness <u>Medications</u>: antibiotics and immunosuppressants	35

Author Year	Age, years	CAP Definition	Comorbidities	Comparison	Additional Covariates	Ref.
Liu 2012	75.2 (10.5)	Diagnostic codes (ICD-9)	Respiratory diseases, DM	The drug exposure immediately prior to the admission for pneumonia ("case" period) was compared to past drug exposure for the same patient ("control" periods)	Number of outpatient visits<br>Medications: statins, H2RA, ACE inhibitors, angiotensin receptor blockers	36
Long 2013	Median 47 (IQR: 35-55)	Diagnostic codes (ICD-9) with antibiotic prescription or hospital admission	Cardiac*, COPD*, DM*, Inflammatory bowel disease (Crohn's or Ulcerative colitis)*, Liver disease*, Renal disease*, Rheumatologic disease*	Matched 1:4 on age, gender, geographic region, type of inflammatory bowel disease, duration of follow-up, and index date using incidence density sampling of IBD patients in same database	Health-care utilization<br>Medications: 5-ASAs, biologics, thiopurine, corticosteroids, narcotics	37
Mastronarde 2009	42 (13)	Not reported	Allergies that worsen asthma, Asthma, Eczema, Food allergies, GERD, Rhinitis, Sinusitis, Tobacco use	Patients in placebo arm of the trial	Medications: inhaled short-acting beta-agonist, oral or inhaled corticosteroids	38
Meijvis 2011	Cases: 62 (18)<br>Controls: 62 (18)	New infiltrate on a chest radiograph with at least two clinical or laboratory findings consistent with pneumonia	Asthma*, CHF*, COPD*, DM*, Renal failure*	Matched 1:4 on birth year, sex, and index date using PHARMO Institute enrollees (research database)	PSI score<br>Medications: Inhaled corticosteroids, anti-cholinergics, NSAIDS, oral corticosteroids	39
Morris 2013	Exposed: 55 (10.7)<br>Unexposed: 63 (14.0)	Positive sputum culture with medical record review for clinical correlation	DM, Kidney transplantation, Hypertension	Unexposed cohort members	None	40
Muellerova 2012	Cases: 75.1 (10.6)<br>Controls: 70.9 (10.8)	Diagnostic codes (Read)	Anxiety, Asthma, CHF, Connective tissue disorder; COPD, CVA, Dementia, Depression, DM, Lung cancer, MI, Peptic ulcer, PVD, Renal disease, Tobacco use	Matched 1:5 on length of follow-up using COPD patients ≥ 45 years old in the General Practice Research Database	None	41

Author Year	Age, years	CAP Definition	Comorbidities	Comparison	Additional Covariates	Ref.
Myles 2009	Cases: >80 (32.5%)<br>Controls: >80 (32.8%)	Diagnostic codes (Read)	Chronic lung disease*, Ischemic heart disease*, Tobacco use*	Matched 1:6 on outpatient practice, sex, and age using The Health Improvement Network enrollees (THIN)	Previous pneumonia, Charlson comorbidity index<br><u>Medications</u>: Diuretics, calcium channel blockers, antacids, steroids & nitrates	42
Nielsen 2012	15-39: 51,601 (7.3%)<br>40-64: 178,095 (25.1%)<br>65-79: 261,046 (36.8%)<br>80+: 218,398 (30.8%)	Diagnostic codes (ICD-8 & ICD-10)	AIDS, Alcoholism-related disorders, Cancer, CHF, COPD, Connective tissue disease, CVA, Dementia, DM, Hemiplegia, Leukemia, Liver disease, Lymphoma, MI, Obesity, Peptic ulcer disease, Peripheral vascular disease, Renal disease, Surgical procedure within 90 days of index date	Matched ratio 1:10 on birth year, sex, index date, and place of residence using community controls selected by risk set sampling of Danish National Registry of Patients	None	43
Pasina 2011	Exposed: 79.2 (7.7)<br>Unexposed: 79.2 (7.5)	Diagnostic codes (ICD-9)	Duodenal ulcer, Esophageal disease, Gastric ulcer, Gastritis, Gastrointestinal hemorrhage, Gastrojejunal ulcer, H. pylori infection, Peptic ulcer	Unexposed cohort members	None	44
Quagliarello 2005	84.7	Compatible symptoms with radiographic findings	Cancer, CHF, COPD*, CVA, Dementia, Depression, DM, Liver disease, Renal disease	Unexposed cohort members	Nursing home site, age, race, number of comorbidities, immobility, inadequate oral care, swallowing difficulty, lack of influenza vaccination, depression, feeding position <90 degrees from horizontal, active smoking<br><u>Medications</u>: sedating medications, ACE-I	45
Ramsay 2013	83 (80-86)	Diagnostic codes (ICD-10)	Not listed	Unexposed cohort members	Age, gender, socioeconomic index of disadvantage for area of residence, number of co-morbidities, number of prescriptions, number of prescribers, number of pharmacies and number of occupational therapy visits and speech pathology services, season, residential aged-care status<br><u>Medications</u>: number of prescriptions, tiotropium, first concurrent use of ARB and furosemide	46

Author Year	Age, years	CAP Definition	Comorbidities	Comparison	Additional Covariates	Ref.
Rodriguez 2009	70-79 (32%)	Diagnostic codes (Read)	Anemia, Asthma, Cancer, Cerebrovascular disease, COPD, Depression, DM, Dyspepsia, GERD, Heart failure, Ischemic heart disease, MI, Peptic ulcer*, RA, Tobacco use*	Frequency-matched on sex, age, and month/year of index date using THIN enrollees	Primary care doctor visits, referrals, hospitalizations, Charlson comorbidity index<br>Medications: oral steroids, antibiotics, H2RA	47
Roughead 2009	79.4 (5.2)	Diagnostic codes (ICD-10)	CHF*, COPD*<br>(via medication proxies)	Unexposed cohort members	Age, sex, socioeconomic index of disadvantage, season, residential age-cared status, occupational therapy visits, RxRisk-V score, speech pathology services<br>Medications: number of prescriptions, prescribers, pharmacies	48
Sarkar 2008	Cases: 73.5 (17.6)<br>Controls: 49.5 (18.3)	Diagnostic codes (Read or Oxford)	Alcoholism*, Cancer*, Cirrhosis*, CHF*, COPD*, CVA, Dementia*, DM*, Dysphagia, MI*, Renal failure*	Matched 1:10 using incidence density sampling of General Practice Research Database enrollees	Age, sex, current smoking status, number of general practice visits, total number of hospitalizations, asthma<br>Medications: H2RAs, anxiolytics, antidepressants, antiparkinson drugs, antipsychotics, barbiturates, opiates, corticosteroids, antibiotics, NSAIDs	49
Scheiman 2011	Exposed: >70 (36.3%)<br>Unexposed: >70 (35.0%)	Not reported	CAD, Duodenal ulcer, Erosive esophagitis, Gastric ulcer, Tobacco use	Placebo arm participants in trial	None	50
Sugano 2011	Exposed: 69.3 (8.57)<br>Unexposed: 68.7 (8.79)	Not reported	Alcohol use, *H. pylori*, Ischemic heart disease, Ischemic stroke, Tobacco use	Gefarnate arm participants in trial	None	51
Sugano 2012	Exposed: 62.8 (11.7)<br>Unexposed: 63.7 (11.1)	Not reported	RA, osteoarthritis, low back pain	Gefarnate arm participants in trial	None	52
van de Garde 2006 (Thorax)	Cases: 80-89 (34.4%)<br>Controls: 70-79 (37.0%)	Diagnostic codes (Read)	Alcohol abuse, CHF*, CVA*, Pulmonary disease*, Tobacco use	Matched 1:4 on age (±2years), gender, General Practioner practice group, and index date using Diabetic General Practice Research Database enrollees	Vaccinations<br>Medications: Statins, current corticosteroids	53
van de Garde 2006 (ERJ)	67 (0.51)	Diagnostic codes (ICD-9)	Respiratory diseases, heart failure, DM	Matched 1:4 on age and sex using unexposed PHARMO database enrollees	None	54

Author Year	Age, years	CAP Definition	Comorbidities	Comparison	Additional Covariates	Ref.
van de Garde 2007 (J HTN)	Cases: 80-89 (34.4%)<br>Controls: 70-79 (37.0%)	Diagnostic codes (Read)	Alcohol abuse, CHF*, CVA*, Pulmonary disease*, Tobacco use	Matched 1:4 on age (±2years), gender, General Practioner practice group, and index date using Diabetic General Practice Research Database enrollees	Vaccinations<br>Medications: Statins, current corticosteroids	55

Age reported as mean (SD) or largest categorical age group (%) unless otherwise noted
* indicates comorbidities included in effect estimate adjustments
Abbreviations: ARB, angiotensin receptor blocker; CAD, coronary artery disease; CAP, community-acquired pneumonia; CHF, congestive heart failure; CKD, chronic kidney disease; COPD, chronic obstructive pulmonary disease; CVA, cerebral vascular attack; DM, diabetes mellitus; FEV1, forced expiratory volume in 1 second; GERD, gastroesophageal reflux disease; H.pylori, Helicobacter pylori; IBD, inflammatory bowel disease; IQR, interquartile range; MI, myocardial infarction; NR, not reported; PVD, peripheral vascular disease; RA, rheumatoid arthritis; Ref, study reference number
