# Supplementary material for: Risk of Community-Acquired Pneumonia with Outpatient Proton-Pump Inhibitor Therapy: A Systematic Review and Meta-Analysis
Source: PLoS One. 2015 Jun 4;10(6):e0128004. doi: 10.1371/journal.pone.0128004 (PMC4456166; doi:10.1371/journal.pone.0128004)
Supplement: S5 Table — (PDF) [file pone.0128004.s009.pdf]

S8 Table. Summary of Sensitivity Analyses

Description	# Cases / # Participants	# of Studies	Pooled Effect Estimate	95% CI	I^2 (%)	p-value for Heterogeneity	Ref.
Adults only*	145358 / 6002023	21	1.36	1.18-1.56	94.4	<0.001	24,25,27,28,31, 32,33,36,38,39, 40,44,45,46,47, 48,49,50,51,52, 54
PPI only†	222715 / 6812317	24	1.48	1.14-1.92	99.2	<0.001	23,24,25,27,28, 31,32,33,35,36, 37,38,39,40,43, 44,46,47,48,49, 50,51,52, 54
Strict CAP definition‡	3733 / 15698	7	1.22	0.99-1.52	65.8	0.007	23,25,28,33,35, 39,45
Low risk of bias§	135030 / 6000720	13	1.26	1.09-1.45	96.3	<0.001	24,25,27,31,32, 33,37,39,45,46, 47,48,49
Adjusted effect estimate	149226 / 6018673	15	1.33	1.16-1.52	95.7	<0.001	24,25,27,28,31, 32,35,36,37,39, 45,46,47,48,49
Observational studies only	222815 / 6809694	22	1.49	1.15-1.93	99.3	<0.001	23,24,25,27,28, 31,32,33,34,35, 36,3739,40,43,4 445,46,47,48,49 , 54

Abbreviations: CI, confidence interval; Ref, study reference number

* Adults only includes studies with more than 95% participants ≥18 years old

† PPI only includes studies with exposure defined as PPI use not grouped with other gastric acid suppressing drugs

‡ Strict CAP definition includes studies that required radiographic confirmation of CAP as part of their case definition

§ Low Risk of Bias includes studies with low risk of bias as determined by defined as low risk on ≥4 out of 7 criteria for cohort studies and ≥6 out of 8 criteria for case-control studies using the modified Newcastle-Ottawa Scale
